# Supplementary material for: Rehybridization dynamics into the pericyclic minimum of an electrocyclic reaction imaged in real-time
Source: Nat Commun. 2023 May 18;14:2795. doi: 10.1038/s41467-023-38513-6 (PMC10195774; doi:10.1038/s41467-023-38513-6)
Supplement: Supplementary file 1 — Supplementary Information [file 41467_2023_38513_MOESM1_ESM.pdf]

SUPPLEMENTARY INFORMATION FOR

REHYBRIDIZATION DYNAMICS INTO THE PERICYCLIC MINIMUM OF AN ELECTROCYCLIC REACTION IMAGED IN REAL-TIME

Y. Liu,[1,2,†] D. M. Sanchez,[1,3,†,‡], M. R. Ware,[1] E. G. Champenois,[1] J. Yang,[1,4,5] J. P. F. Nunes,[6,7] A. Attar,[4] M. Centurion,[6] J. P. Cryan,[1] R. Forbes,[4] K. Hegazy,[1] M. C. Hoffmann,[4] F. Ji,[4] M.-F. Lin,[4] D. Luo,[4] S. K. Saha,[6] X. Shen,[4] X. J. Wang,[4,] T. J. Martínez,[1,3,*] & T. J. A. Wolf[1,*]

[1]Stanford PULSE Institute, SLAC National Accelerator Laboratory, 2575 Sand Hill Road, Menlo Park, CA 94025, USA.

[2]Department of Physics and Astronomy, Stony Brook University, Stony Brook, NY 11790, USA.

[3]Department of Chemistry, Stanford University, 333 Campus Drive, Stanford, CA 94305, USA.

[4]SLAC National Accelerator Laboratory, 2575 Sand Hill Road, Menlo Park, CA 94025, USA.

[5]Center of Basic Molecular Science, Department of Chemistry, Mong Man Wai Building of Science and Technology, S-1027 Tsinghua University, Beijing, China.

[6]Department of Physics and Astronomy, University of Nebraska-Lincoln, Theodore Jorgensen Hall 208, 855 N 16th Street, Lincoln, NE 68588, USA.

[7]Diamond Light Source, Harwell Science Campus, Fermi Ave, Didcot OX11 0DE, UK.

*Corresponding authors. Emails: thomas.wolf@slac.stanford.edu, toddjmartinez@gmail.com

† These authors contributed equally to this work.

‡Present address: Design Physics Division, Lawrence Livermore National Laboratory, Livermore, USA.

TABLE OF CONTENTS

SUPPLMENTARY NOTES 2

Note 1, Signatures from Hydrogens in Pair Distribution functions 2

Note 2, Structural Signatures of αTP Rotamers 3

Note 3, Error Function Fits to Temporal Onsets of the ΔPDF Signal in regions α, β, and γ.......4

Note 4, Conrotatory Hydrogen Motion..4

Note 5, Conrotatory Planarization and Deplanarization Motions..5

Note 6, Ultrafast electron diffraction signal processing 5

Note 7, αTP Photoinduced Ring-Opening Dynamics 11

FIGURES 15

TABLES 34

SUPPLEMENTARY REFERENCES 36

SUPPLMENTARY NOTES

Note 1, Signatures from Hydrogens in Pair Distribution functions

The contributions from (C,H) and (H,H) distances to the static PDF of αTP are considerable smaller than those from (C,C) distances, but not negligible (see Supplementary Figure 4, top). However, as we show at the example of a simulated ΔPDF based on an optimized photoproduct and a reactant geometry (Supplementary Figure 4, bottom), The contributions from (C,C) and (C,H) distances to transient signatures are considerably smaller than to static signatures. This effect can be explained by the fact that the hydrogens closely follow the carbons, to which they are bound.

Note 2, Structural Signatures of αTP Rotamers

αTP exhibits a total of three ground state conformers, which differ by the rotation of the isopropyl group around the bond connecting it to the ring (M, P, and T in Supplementary Figures 5 and 6). Supplementary Figure 5 shows the simulated PDFs as well as the carbon coordination sphere contributions for each of the rotamers compared to experiment. The most significant difference between the experimental and simulated PDFs in Supplementary Figure 6a is the magnitude of the minimum at 3.5 Å, which is connected to the bimodal distribution in the third carbon-carbon coordination sphere. The reasons for these differences are differences in orientation of the (C_7) and (C_8) carbon atoms with respect to the ring plane. In the M and P rotamers, one of the carbons is oriented in the ring plane and the other one in approx. 120° angle to the ring plane. In the T-rotamer, both carbons are oriented with an absolute angle of approx. 120° degree with respect to the ring plane. Thus, for the M and P rotamers, only third coordination sphere distances related to one of the two carbons fall in the center of the third coordination sphere distance range. For the T rotamer, distances related to both carbons fall into the center of the third coordination sphere range. This results in a deeper minimum for M and P rotamers than for the T rotamer. This feature can be reviewed from Supplementary Figure 6b, where two small peaks display in the center region of the 3rd coordination sphere in rotamer T. The fact that the minimum depth of the experimental PDF falls in between the m/p and t rotamer values suggests that our sample consisted of a thermal equilibrium of the three rotamers.

In addition to the static ground state PDFs, Supplementary Figure 7 shows the computed time-dependent ΔPDF for each rotamer from the excited-state dynamics and compared directly to the measured signal. Panels a, b and c reflect the signals of M, P, and T, respectively. Panel d shows a lineout of the signals at a delay around 550 fs among the three rotamer and the measured

signal. In general, the rotamers undergo similar dynamics and the amplitudes of the peaks and troughs match well. According to panel d, ΔPDFs of the rotamer m/p are almost identical and the main difference is from t at the pair distance between 3 and 5 Å. However, the difference from the different rotamers cannot be distinguished from the experimental measurement.

Note 3, Error Function Fits to Temporal Onsets of the ΔPDF Signal in regions α, β, and γ

We characterize the temporal onset of the integrated ΔPDF Signal the α, β, and γ regions by fitting an error function

$$\Delta PDF(t) = A + B \cdot erf\left(2 \cdot \sqrt{\ln(2)}\,\frac{t - t_0}{\tau}\right)$$

to it, where A is a constant offset, B the signal amplitude, t_0 the center of the error function, and τ a measure for the width of the error function. In Fig. 3b, we show the parameter values of t_0 and τ from fits to the experimental data as a point with an error bar representing the uncertainty of the fit and as a colored bar, respectively. Additionally, we show a comparison of the fits to experimental data and simulations in Supplementary Figure 8. The fitted values for t_0 and τ are listed in Supplementary Table 1.

Note 4, Conrotatory Hydrogen Motion

The evolution of the excited state wavepacket of αTP within the first 80 fs in conrotatory movement of the hydrogens around the (C_3) and (C_4) carbons is depicted in Supplementary Figure 9 (coordinate defined in the caption and in Ref.[1]). For comparison, also the projection onto the complementary disrotatory coordinate is shown. The wavepacket motion has significantly stronger conrotatory than disrotatory character. Additionally, the conrotatory motion is Franck-Condon active. However, its amplitude is only on the order of 1 degree, which points to the presence of the

(C_3-C_4) σ bond constraining CH_2 rotation. Moreover, the timescale of the motion does not coincide with internal conversion or (C_3-C_4) bond dissociation.

Note 5, Conrotatory Planarization and Deplanarization Motions

The amplitudes of the individual planarization motions (green and purple curves in Fig. 4b) show close to identical amplitudes, whereas the corresponding deplanarization motions in Fig. 4c show a stronger amplitude for the deplanarization motion involving the methyl group. The behavior is connected to the much larger moment of inertia of the isopropyl substituent compared to the methyl substituent. Thus, the planarization motion around the (C_3=C_1) double bond is dominated by motion of the (C_3) hydrogens, whereas the methyl substituent is more strongly involved in the corresponding motion around the (C_4=C_5) double bond. This observation is in line with our interpretation of the early onset of the ɣ as being dominated by out-of-plane motion of the methyl group.

Note 6, Ultrafast electron diffraction signal processing

Diffraction Signal Treatment: After the experimental data acquisition, each measured diffraction pattern undergoes a quality evaluation and control routine which consists of several processes including, 1) signal baseline subtraction, 2) rejection of images with low signal intensity, 3) removal of "hot" pixels, and 4) median filtering and normalization. More details of the analysis procedure of the signal treatment can be found in the supplementary materials of Ref.[2–5] After the evaluation and control process, the diffraction centers of each individual image are located. The center of each image is determined by a least square fitting algorithm on the diffraction pattern.

To account for the changes in the total electron number in each pulse, each diffraction pattern (or image) is normalized to the total signal in the ranges of $2 < s < 8$ Å^{-1}.

Diffraction Percentage Difference: We optimize our analysis of the experimental data by evaluating diffraction percentage difference signals. The 1-dimensional scattering intensity as a function of s, $I_{exp}(s)$, is obtained by azimuthally averaging the 2-dimensional diffraction pattern using the determined centers. To highlight the time-dependent changes in the data, we calculate the percentage difference signal according to the following equation,

$$\%\Delta \frac{I}{I_{exp}}(s,t) = \frac{I_{exp}(s,t) - I_{exp}(s,t<0)}{I_{exp}(s,t<0)} \times 100 \quad \textbf{(2)}$$

where $I_{exp}(s,t)$ is the total scattering intensity for each pump-probe delay time, t. The reference signal $I_{exp}(s,t<0)$ is obtained by averaging the diffraction signal measured at delays -2 ps < t < -500 fs, which corresponds to the static, unpumped scattering signal. Supplementary Fig. 10 shows a false-color plot of the experimental $\%\Delta \frac{I}{I_{exp}}(s,t)$ signal. Supplementary Figure 11 panels a and b show several representative delay-slices of the measured signals (curves) and uncertainty (shaded areas) at specific pump-probe delays before and after a further background removal process, respectively. In this process, a low-order (up to 2nd order) polynomial is fitted over the whole s range.[4,6] This procedure has little effect on the low s region, but significantly helps to reduce noise and systematic offsets at high s (see Supplementary Figure 11). We applied a standard bootstrapping analysis to estimate the statistical uncertainty of the measurement. In total, the data set contains 145 runs, and a single run contains diffraction patterns at each individual delay. We made use of these 145 runs to create bootstrapped datasets. In each bootstrapped dataset, 145 diffraction patterns are randomly selected per delay step and this process is repeated by 150 times, generating 150 bootstrapped datasets. Each dataset is analyzed along the same protocols such that

a mean and standard deviation can be evaluated for the variables with interests. Supplementary Figure 11 panel b shows the error bar of the measurement which is one standard deviation of the $\%\left(\frac{\Delta I}{I}\right)_{exp}$ from the bootstrapping analysis. The amplitude of the error bar is roughly the same order of the noise level before time-zero, which is significantly smaller than the signal level in the positive delays. Supplementary Figure 12 reflects the uncertainty development as a function of the number of bootstrapped datasets used in the analysis. The uncertainty quickly develops in the first 50 bootstrapped datasets, with larger amplitude changes in both standard deviation $\sigma_{(\%\Delta I/I)}$ and the standard deviation of the mean saturation. However, both start saturating after running 150 bootstrapped datasets.

Excitation Ratio and Time-Zero Location: We estimate the experimental excitation ratio by comparing experimental and simulated diffraction signals at long delay times. Supplementary Figure 13 shows the measured $\%\left(\frac{\Delta I}{I}\right)_{exp}(s,t)$ versus the simulated diffraction percentage difference, $\%\left(\frac{\Delta I}{I}\right)_{theory}(s,t)$, averaged between 0.8 and 1.2 ps. By multiplying a factor of 0.0156, the experimental data and simulation match well. Thus, we use this value (γ = 1.56 %) as an estimation of the excitation ratio in the experiment.

During the experiment, we obtain time zero with the help of ultrafast heating heating from a solid-state bismuth target. However, other than in time-resolved electronic spectroscopy, the onset of such a heating signal never coincides precisely with time zero, since the nuclei have to move considerably to show a transient effect. Therefore, the experimental time zero must be corrected for a quantitative comparison with simulations. This can be achieved with the help of the simulations themselves if the agreement between experiment and simulation is sufficient. Thus, the exact time zero of the experimental data is determined by comparing the the time dependence

of the integrated signal in a window between 2.8 and 3.5 Å^{-1} of $\%\left(\frac{\Delta I}{I}\right)_{theory}(s,t)$, broadened with a 150 fs FWHM Gaussian to account for the limited time resolution of the experiment, with $\%\left(\frac{\Delta I}{I}\right)_{exp}(s,t)$ integrated in the same window. We use a least-square fit to overlap the resulting signals. The fit results are shown in Supplementary Figure 14. The best fit yields a shift of about 105 fs.

Pair Distribution Functions: After the diffraction pattern analysis, we calculate the time-dependent atomic pair distribution functions (PDFs) following standard procedures that were developed by Zewail and co-workers and we made use of PDFs to extract the structural information.[6–8] Instead of using the percentage difference in Eq. 2, we utilized the modified scattering intensity, $sM(s;t)$, to generate the map of the $PDF(r,t)$. We calculate the $\Delta sM(s,t)$ by following the equations below,

$$\Delta sM_{exp}(s,t) = \frac{I_{exp}(s,t) - I_{exp}(s,t<0)}{I_{ato}(s)} s \quad \textbf{(3)}$$

$$I_{ato}(s) = \sum_{i=1}^{N} f_i^*(s) f_i(s) \quad \textbf{(4)}$$

where $\Delta sM_{exp}(s,t)$ is the delay dependent modified scattering intensity, $I_{ato}(s)$ the atomic scattering, $f_i(s)$ is the scattering amplitude of the ith atom calculated using the ELSEPA program.[9] The ΔPDF of the measurement is then obtained by applying a sine transform of the $\Delta sM_{exp}(s,t)$ following the equation below:

$$\Delta PDF_{exp}(r;t) = \int_0^{s_{max}} \Delta sM_{exp}(s,t) sin\,(sr)\, e^{-\kappa s^2} ds \quad \textbf{(5)}$$

where r represents the pair distance in real space, κ is the damping constant and s_{max} the magnitude of the largest scattering vector that the detector can accommodate. To minimize noise and artifacts at larger s values, Eq. 5 includes a Gaussian damping function $e^{-\kappa s^2}$ which smoothly reduces the intensity of the measured signal towards zero at the larger s region. Here we use κ=

0.03, which corresponds a Gaussian function with half-width-half-maximum (HWHM) ≈ 7 $Å^{-1}$. Supplementary Figure 1 showcases the experimental and simulated ΔPDFs. The integration ranges for the signatures in Fig. 3b of the main text are shown in Supplementary Figure 15.

Low S Diffraction Signal Treatment: A general issue in the real space transformation of diffraction signals is the missing signal in the low s region which is due to the hole in the center of the phosphor screen detector. Extrapolation of the signal to $s = 0$ $Å^{-1}$ is required to avoid artifacts in the $\Delta PDF_{exp}(r,t)$. We fill in the missing experimental signal with the simulated signal following the equation below,

$$\Delta sM_{exp}(s < s_{hole}, t) = \beta(t)\Delta sM_{simu}(s \leq s_{hole}, t = 1\,ps) \quad \textbf{(6)}$$

$$\beta(t) = \gamma\left(1 + \left(\frac{t - t_0}{\tau}\right)\right) \quad \textbf{(7)}$$

Eq. 6 consists of two terms, in which $\Delta sM_{simu}(s \leq s_{hole};\ t = 1\,ps)$ is the average of the simulated ΔsM in a window between 0.98 and 1 ps in the relevant low s range. The second term, $\beta(t)$, sets the time-dependent amplitude of the simulation, which is constrained to follow a simple error function with the onset time t_0 and width τ found by curve fitting to ΔsM_{exp} at $s > s_{hole}$. By multiplying the excitation ratio, γ, the second term fulfills the amplitude matching of the small s signal. Supplementary Figure 1a showcases the measured ΔPDFs after the treatment of the low S signal discussed above. Supplementary Figure 16 shows an example of the influence from the missing data in $s < 0.8$ $Å^{-1}$. Panels a and b show the $\Delta sM(s)$ and corresponding $\Delta PDF(r)$ from the simulation with or without the low s region substituted by simply zeros, respectively. From panel **b,** the main artifacts induced by the substitution of zeros in the low s region are a global tilted offset across the whole pair distance range in the real space, with a smooth positive contribution to pair distance < 3 Å and a smooth negative contribution > 4 Å. Panels c and d reflect the measured and simulated time-dependent $\Delta PDF(r,t)$ after the substitution of the low s signal

with zeros. Compared with the signals in Fig. 2 of the main text and Supplementary Figure 1a, it is clear that the artifact below 2 Å from the experiment (panel c in Supplementary Figure 16) is much stronger, and the additional positive signal in the simulation (panel d in Supplementary Figure 16) which is not shown in Fig 2.

Simulated Diffraction Signals: In the simulated diffraction signal, the modified difference diffraction signal defined above, $\Delta sM(s,t)$, is generated from the AIMS/DFT trajectories using the independent atomic model (IAM) and converted to $\Delta PDF(r,t)$ using identical code and procedures as for the experimental data. The total diffraction signal, $I_{Mol}(s,t)$, is computed as an average over all 60 ICs (174 TBFs), where the diffraction signal for a specific IC is approximated as an incoherent sum over weighted diffraction signals from individual TBFs:

$$I_{Mol}(s,t) = \frac{1}{N_{IC}} \sum_{M=1}^{N_{IC}} \sum_{k}^{N_{TBF}^{M}(t)} n_k^M(t) I_{Mol}^{k,M}(s,t) \quad \textbf{(8)}$$

where N_{IC} is the number of ICs, $N_{TBF}^{M}(t)$ is the number of TBFs at time *t* for the *M*th IC, $n_k^M(t)$ and $I_{Mol}^{k,M}(s,t)$ are the weight and diffraction signal for the *k*th TBF of the *M*th IC at time *t*, respectively. The expression for $I_{Mol}^{k,M}(s,t)$ is identical to that used in the experimental diffraction signal, augmented with a Gaussian factor to account for the finite width of the TBFs:[10]

$$I_{Mol}^{k,M}(s,t) = \sum_i \sum_{j\neq i} |f_i(s)| \cdot |f_j(s)| cos(\eta_i - \eta_j) \frac{sin(s \cdot R_{ij}(t))}{s \cdot R_{ij}(t)} e^{-(\alpha_i^2+\alpha_j^2)s^2} \quad \textbf{(9)}$$

where α_i and α_j represent the finite widths for the atoms used in the TBFs.[10] These widths are taken to be element specific and are 0.112 Å/0.249 Å for carbon/hydrogen. The atomic form factors, $f_i(s)$, are calculated from ELSEPA. R_{ij} is the interatomic distance between the *i*th and *j*th atoms taken from the centroids of the AIMS/DFT TBFs. The weight $n_k^M(t)$ is evaluated according to the bra-ket averaged Taylor expansion (BAT) method:[11]

$$n_k^M(t) = \frac{1}{2} \sum_{l}^{N_I^M(t)} [c_k^*(t) S_{kl} c_l(t) + c_l^*(t) S_{lk} c_k(t)] \quad \textbf{(10)}$$

The complex amplitudes are time-independent during the DFT adiabatic dynamics and are held constant at the value from the last frame of their corresponding AIMS trajectory. This is valid because the ground state TBFs are effectively uncoupled from all other TBFs.

The theoretical $\Delta sM(s,t)$ analog to the experimental diffraction signal is computed by subtracting the static diffraction of αTP from all 2 fs time-bins. In the case of the simulations, this is the diffraction of the initial conditions, $\Delta sM(s,t=0)$. The ΔsM(s,t) is convolved with a 150 fs full width at half maximum temporal Gaussian to match the experimental instrument response function. We use the same scripts as for the experimental data to generate $\Delta PDFs$. The error bars in Fig. 3 of the main text represent the standard deviation of the ΔPDF values as evaluated by bootstrap sampling from the initial conditions. They are a measure for the level of convergence of the simulation for the employed number of ICs.

Note 7, αTP Photoinduced Ring-Opening Dynamics

Excited State Dynamics on S_1: The photoinduced ring-opening of αTP follows very closely to its parent molecule, CHD. Like CHD, the S_1 and S_2 adiabats in the FC region of αTP exhibit the diabatic character of a single and double electron excitation from its highest occupied molecular orbital (HOMO) to its lowest unoccupied molecular orbital (LUMO), respectively (see CI coefficients in min S_0 in the Supplementary Data). The S_1 adiabat changes character as the wavepacket evolves away from the FC region (See CI coefficients in S_0/S_1 MECI in the Supplementary Data), implying there is a CI between S_2 and S_1. As we have shown in our previous work, the S_2/S_1 CI is considerably sloped and will be almost avoided entirely by the wavepacket as αTP evolves towards the S_0/S_1 CI. Therefore, we are confident that αTP's relaxation mechanism can be described in its entirety within two adiabatic states. For this reason, all three rotamers of

αTP were placed on S_1 to simulate its photoinduced ring-opening and the subsequent ground state isomerization dynamics in isolation for the first 1 ps.

All three rotamers of αTP show essentially identical photoinduced ring-opening dynamics and resemble CHD quite closely. Supplementary Figure 5a shows the population dynamics of all three rotamers as TBFs are spawned and population is transferred from S_1 to S_0. The decay constant for the photoinduced ring-opening process is 168 +/- 22 fs when averaged over all 60 ICs, which is slightly slower than CHD (139 +/- 25 fs) and faster than αPH (~456 +/- 115 fs (axial) and 285 +/- 71 fs (equatorial)). The orientation of the isopropyl group does not seem to influence the decay time (Supplementary Figure 5a). Furthermore, Supplementary Figure 5b shows that population transfer events mainly take place in the first 200 fs of dynamics when the C_3-C_4 distance is elongated past 1.8 Å as the wavepacket traverses through the S_0/S_1 CI. Supplementary Figure 5c shows that the majority of spawning geometries are quite similar in structure and energy (within 0.1 eV) to the ring-open MECI. Therefore, we treat all rotamers on an equal footing when computing observables from the AIMS dynamics.

αTP strictly relaxes through the ring-opening nonradiative relaxation pathway via a conrotatory fashion. Supplementary Table 2 shows the branching ratio from all 60 ICs between the closed- and open CIs, with effectively 100% of the wavepacket reaching the S_0/S_1 CI. This allows us to directly follow the conrotatory and disrotatory ring-opening motion for the entire wavepacket. Supplementary Figure 9 shows the projection of the wavepacket population onto conrotatory and disrotatory angles (see Supplementary Figure 9 inset) for the first 80 fs of the excited state dynamics. Increasing conrotatory/disrotatory angle means the ring opens in a conrotatory/disrotatory fashion. After excitation, the conrotatory angle increases dramatically, while the disrotatory angle remains relatively small. When the wavepacket reaches the S_0/S_1 CI, it

can either return to the photoreactant αTP or go on to form the photoproduct cZc IPMHT. As is the case with CHD, the S_0/S_1 CI is relatively peaked for all rotamers of αTP (Supplementary Figure 17). Supplementary Table 2 shows that approximately 42 +/- 4 % of the wavepacket results in the formation of αTP while 58 +/- 4 % forms cZc-IPMHT. This relatively split branching ratio can be attributed to the peak-like of the S_0/S_1 CI. Within the bootstrapped error bars, we found no single rotamer to be remarkably different.

Ground State Dynamics on S_0: The natural evolution of the wavepacket dynamics on the ground state can be followed by binning geometries along ground-state TBFs into one of the αTP photoproducts (Fig. 1). Following previous studies,[3,5] snapshots taken every 50 fs along all 174-ground state TBFs were binned into one of the isomers based on dihedral angles (Φ1, Φ2, and Φ3) and atomic distance C_3-C_4 (R_1) (Supplementary Figure 18) Due to the ground state TBFs being sufficiently uncoupled from all others, Eq. 10 can be reduced to:

$$n_k^M(t) = |c_k^M(t)|^2 \quad \textbf{(11)}$$

where the total population of a specific isomer L at time t on the ground-state, $P_L(t)$ is computed by:

$$P_L(t) = \frac{1}{N_{IC}} \sum_{M=1}^{N_{IC}} \frac{\sum_k^{N_{TBF}^M(t)} c_{k,M}^*(t)\, c_{k,M}(t)\, \delta\left(L, I\left(\underline{R}_{k,M}(t)\right)\right)}{\sum_k^{N_{TBF}^M(t)} c_{k,M}^*(t)\, c_{k,M}(t)} \quad \textbf{(12)}$$

where L is defined as (cZc, tZc/cZt, tZt, and αTP), $c_{k,M}(t)$ is the amplitude of the *k*th TBF and *M*th IC, $N_{IC}$is the total number of initial conditions, and $\delta(\ldots)$ is a Kronecker-delta function, and $I(R)$represents the isomer classification of the geometry given by **R**.

Supplementary Figures 19a-d show how the excited state population relaxes into different ground-state isomers via the ring-open CI. Following the total wavefunction from all 60 ICs (Supplementary Figure 19a), nearly all of the ground state population is either αTP or cZc-IPMHT

in the first 150fs after photoexcitation. Like the individual rotamers in Supplementary Figures 19b-d, the cZc-IPMHT population increases slightly quicker when compared to αTP, but quickly decreases upon the formation of the cZt/tZc-IPMHT isomers. In all panels, the cZt/tZc-IPMHT isomers are initially short lived, as the terminal ethylene groups in the "hot" ground state mixture quickly isomerize to form tZt-IPMHT. However, we only observe a large tZt-IPMHT peak initially, with the steric clash from the inward facing isopropyl group limiting its formation at later times. Similar to CHD and αPH, a small portion of the population is effectively "trapped" in cZt/tZc-IPMHT with each subsequent transformation between cZc and tZt-IPMHT. Lastly, the αTP population gradually approaches a limit between 40-50%, where the population is unable to overcome the large ground state barrier separating αTP and cZc-IPMHT.

FIGURES

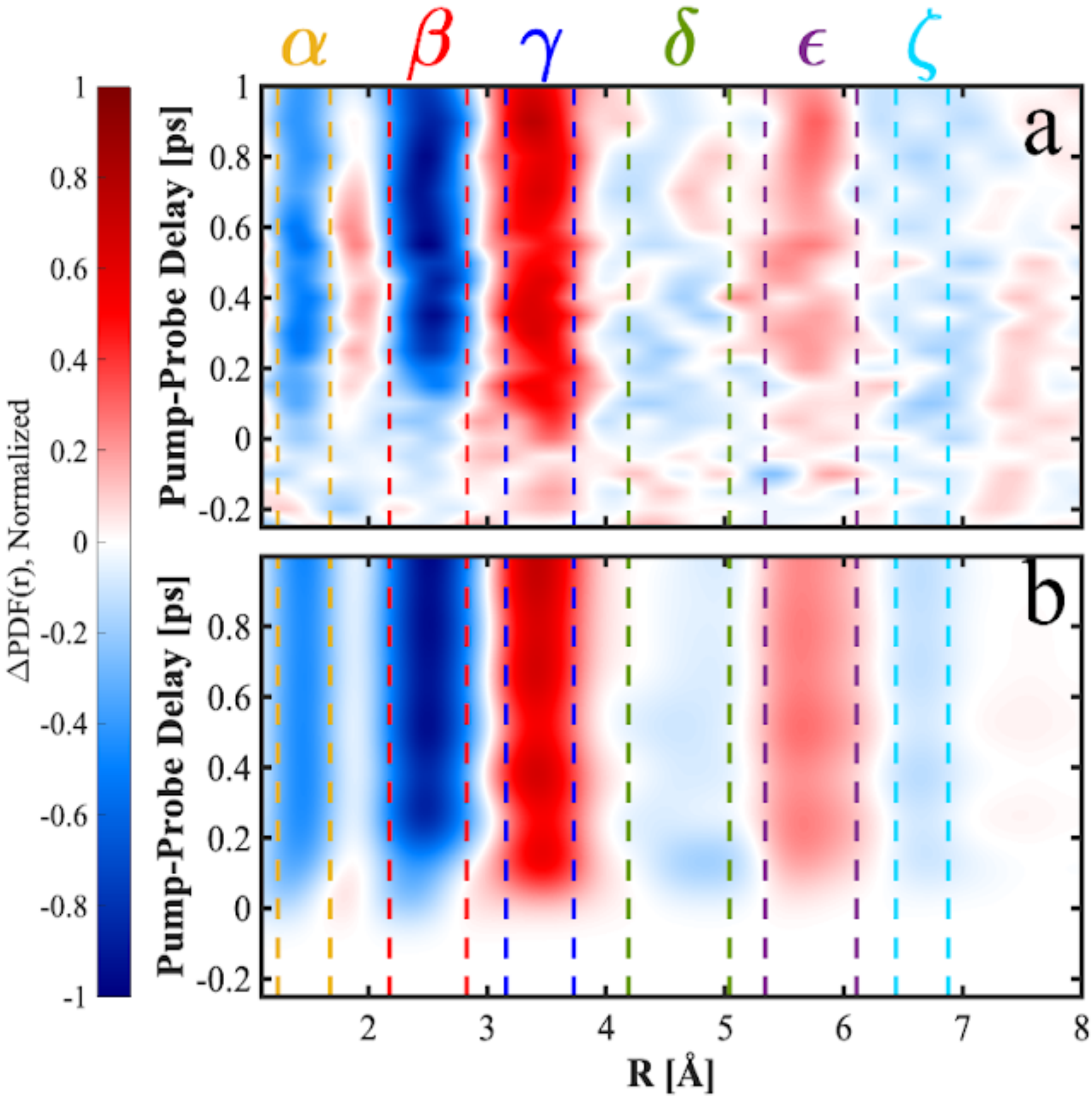

Supplementary Figure 1. Experimental (a) and simulated (b) ΔPDF for the first 1 ps of αTP photoinduced ring-opening dynamics. All difference features (positive peaks and negative troughs) are identified by the letters α to ζ.

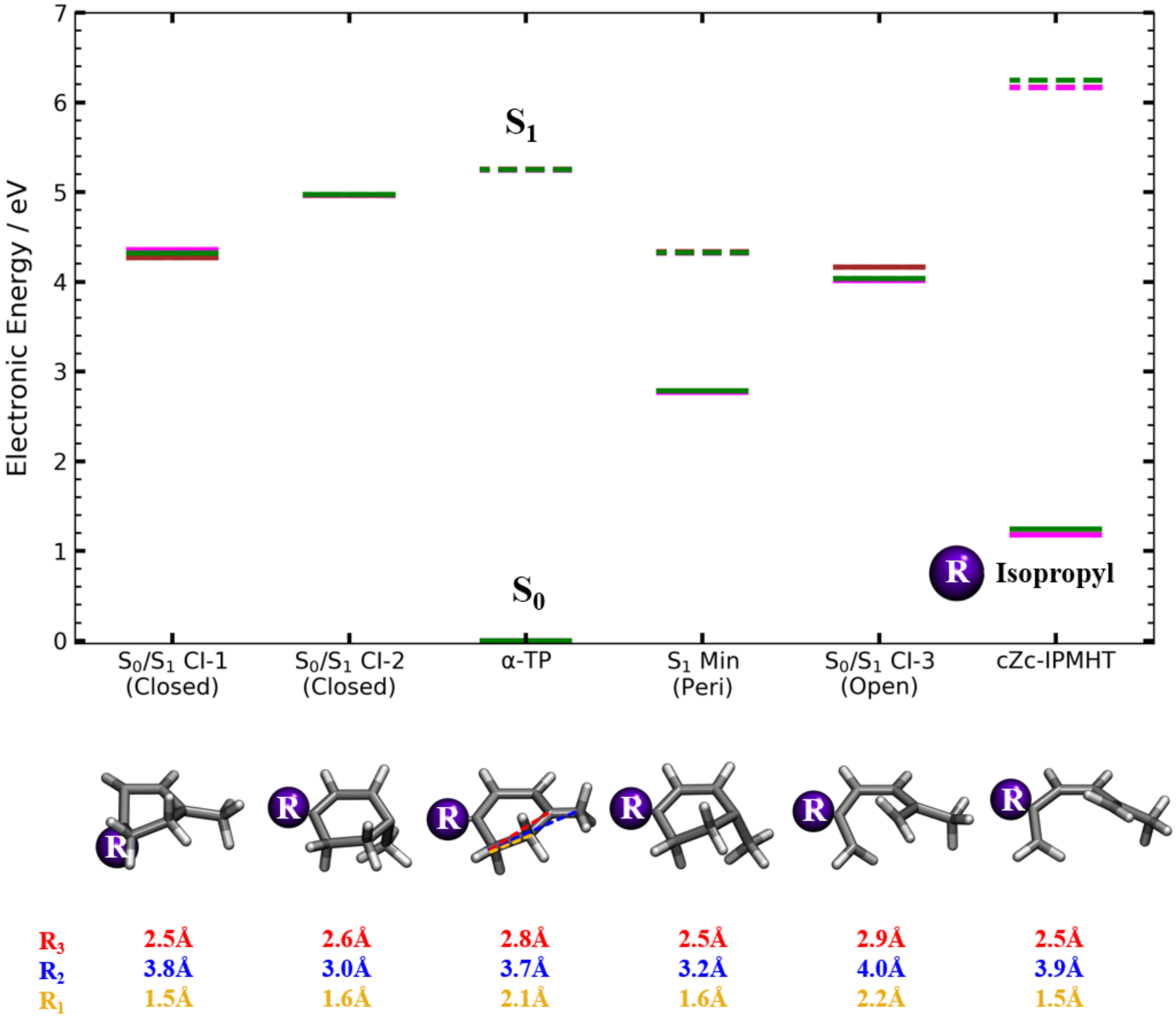

Supplementary Figure 2. Critical Points Along Nonradiative Relaxation Pathways in αTP. The energies are relative to each rotamer's respective S_0 minimum ground-state energy (rotamer M (red), rotamer P (pink), and rotamer T (green)). Geometries shown in the bottom of the figure with the purple spheres representing the location of the isopropyl are computed at the α (0.82)-SA2-CAS(6,4)-SCF/6-31G* level of theory. See **Supplementary Data** for Energies, Cartesian Coordinates, and CI Vectors.

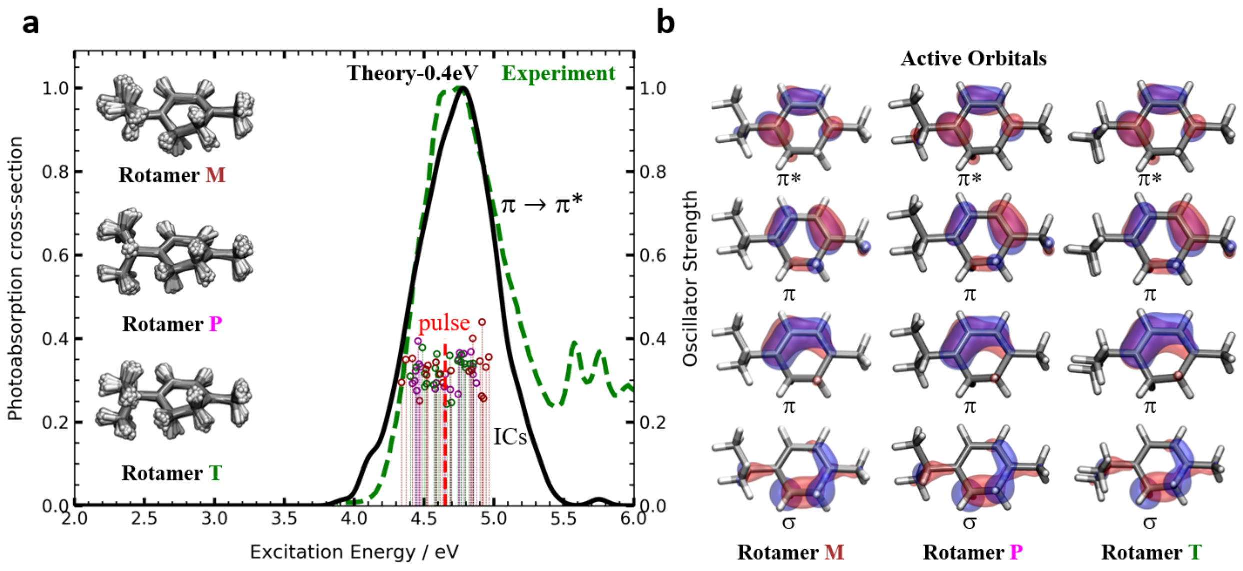

Supplementary Figure 3. UV Electronic Absorption Spectrum of αTP. The computed UV electronic absorption spectrum (black) was generated from 600 initial conditions sampled from a ground-state harmonic Wigner distribution and compared against experiment (green). The AIMS dynamics simulations used 20 initial conditions for each rotamer (M (red), P (pink), T (green)). In panel a, the energy and oscillator strength for each of the initial conditions (randomly sampled with the restriction that they were within 0.3 eV of the pump pulse energy used in the UED experiment) are shown with red/pink/green vertical lines for the M/P/T initial conditions, respectively. The inset shows the starting geometries for each rotamer examined in this study. Panel b shows the SA-CASSCF natural molecular orbitals of the (6,4) active space at the S_0 minima of the three different rotamers. Blue and red correspond to 0.05 and -0.05 $e^-/Å^3$ isovalues, respectively.

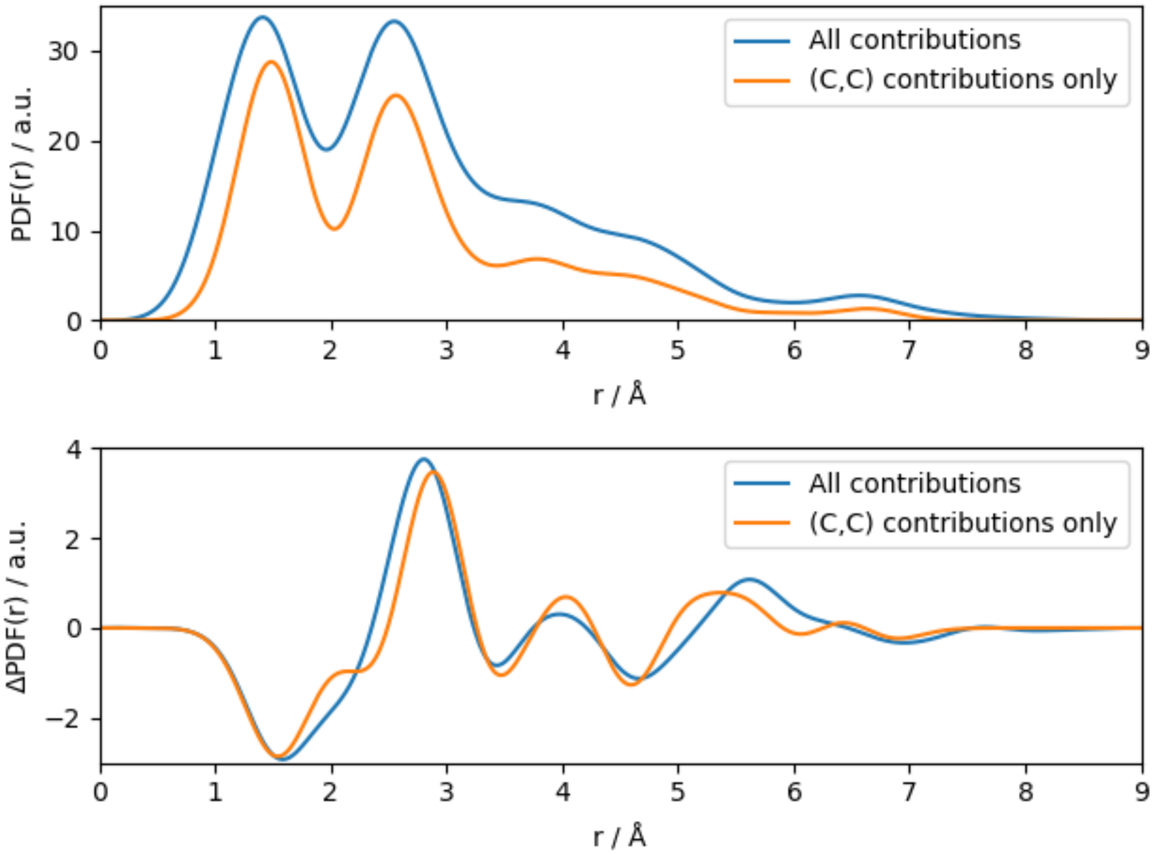

Supplementary Figure 4. Contributions from C-H and H-H distances to pair distribution functions (PDF) and difference pair distribution functions (ΔPDF). Top: Comparison of simulated PDFs of αTP including all contributions and (C,C) contributions only. Bottom: Analogous simulations of ΔPDF of the cZc photoproduct isomer of αTP.

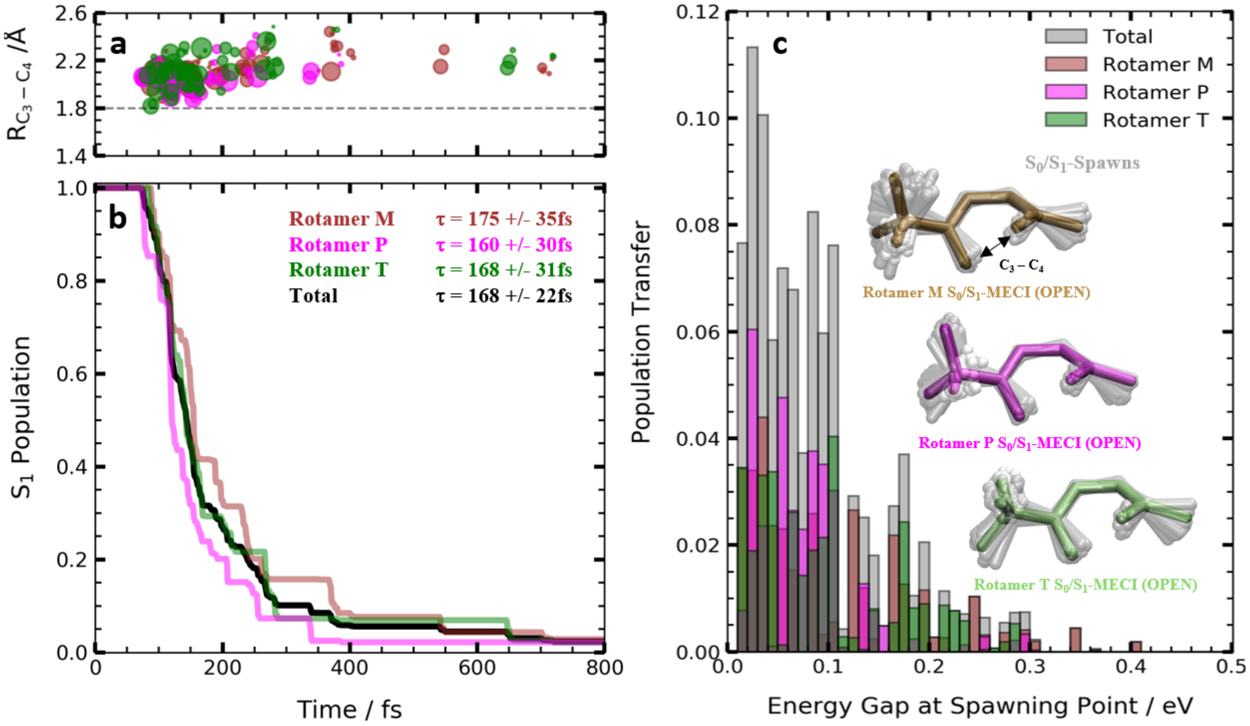

Supplementary Figure 5. Details from the AIMS simulations. a) The C_3-C_4 bond distance vs spawning time at the spawning geometries from the AIMS trajectories for rotamers M (red), P (pink), and T (green). The circle radius is proportional to the population transferred during the spawning event. The population transfer is defined as the total population transferred to a child TBF from the beginning of coupled propagation until the child TBF becomes completely uncoupled (off-diagonal elements in the Hamiltonian become small). **b**) The population of the wavepacket on the S_1 adiabat for the first 800 fs of the photodynamics of αTP. Decay constants from single-exponential fits along with bootstrapped errors are shown in the inset. **c**) Histogram of the population transfer vs the energy gap of all S_1/S_0 spawning geometries from the AIMS simulation for all three rotamers. Spawning geometries superimposed on their respective ring-opening MECI are shown in the inset with all hydrogens (except for the isopropyl) removed for clarity.

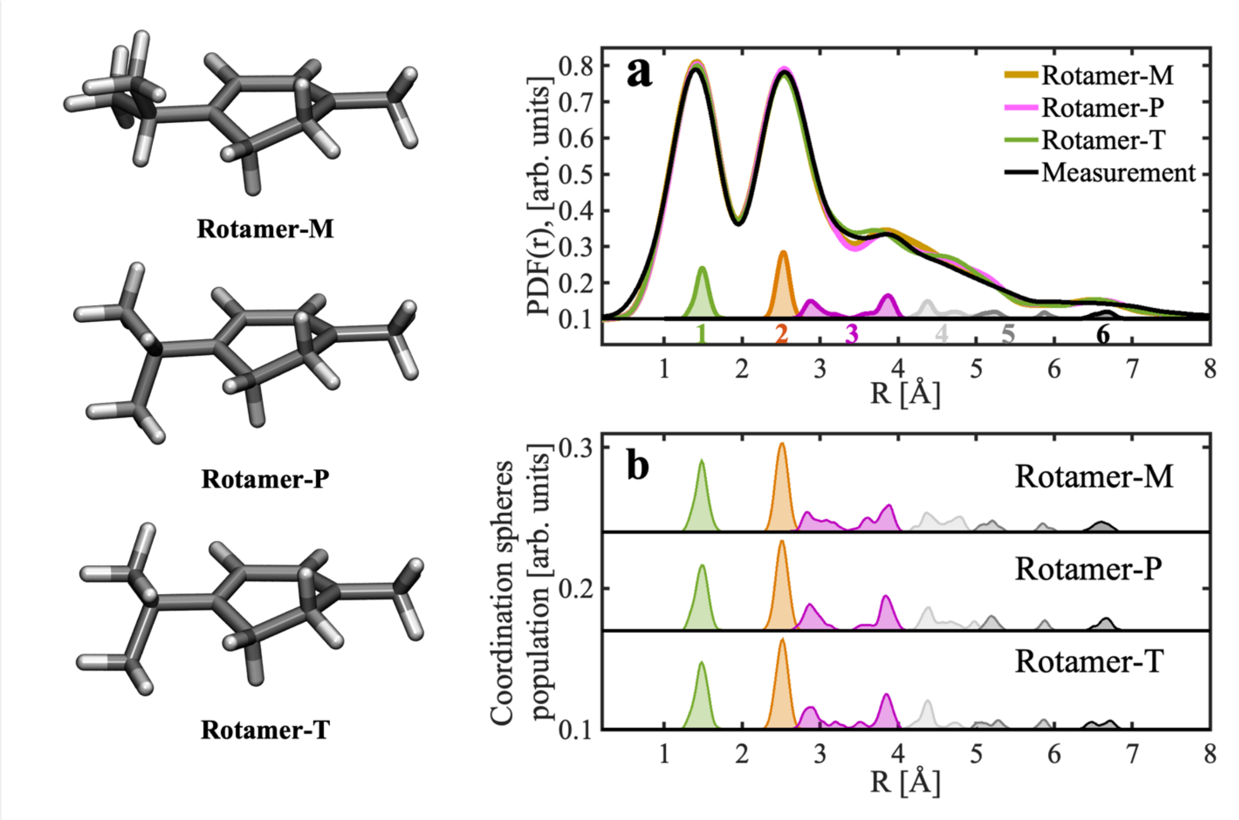

Supplementary Figure 6. Pair distribution functions of αTP ground state rotamers. Panel **a** plots both the experimental and simulated ground state pair distribution functions. The simulated PDF(r) include all the three rotamers. The difference of PDF(r) between rotamer M and P is rather small and only rotamer T displaces certain amount of difference at the 3rd and 4th coordination shells. The colored shaded areas underneath the PDF curves reflect the relevant contribution from carbon-carbon atomic pairs in each different carbon coordination spheres from all the three rotamers. The shading plots in panel **b** reflect the contribution of carbon coordination spheres from each individual rotamers.

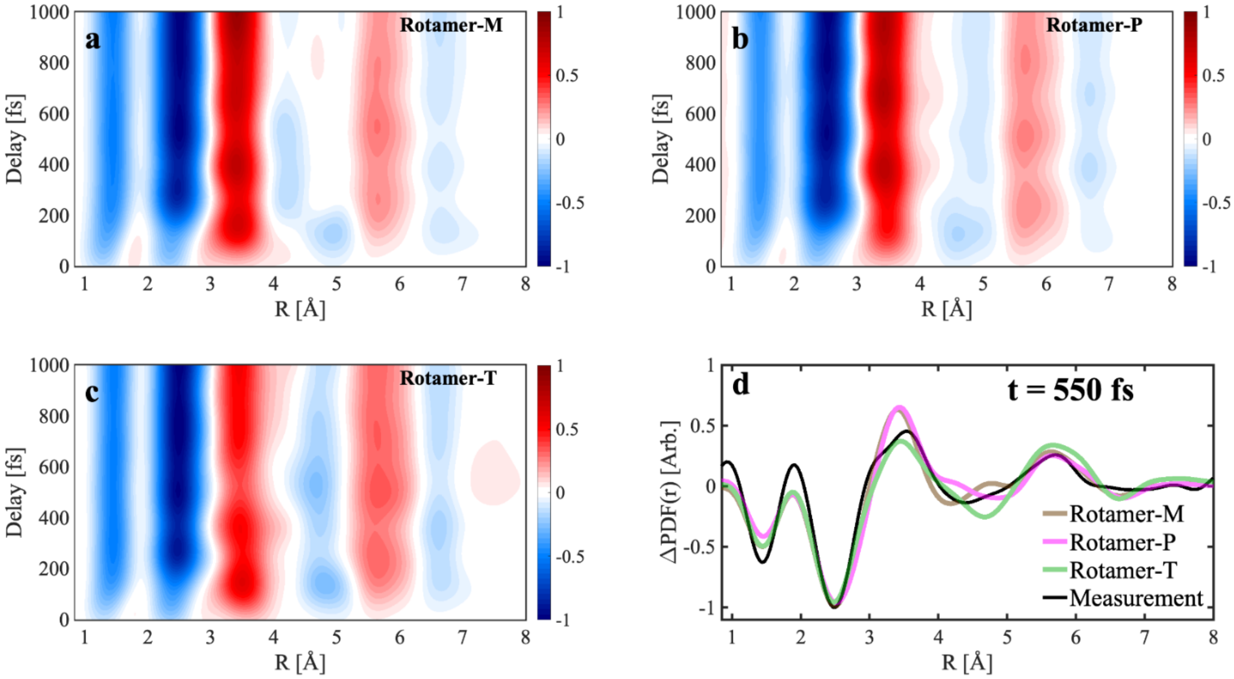

Supplementary Figure 7. ΔPDF(r, t) from three rotamers of α-terpinene. Panels **a**, **b**, and **c** reflect the time-dependent ΔPDFs from the AIMS trajectories of rotamers M, P and T, respectively. Signal in each panel is averaged from 20 initial geometries. Panel **d** shows the ΔPDFs at t = 550 fs of the three rotamers.

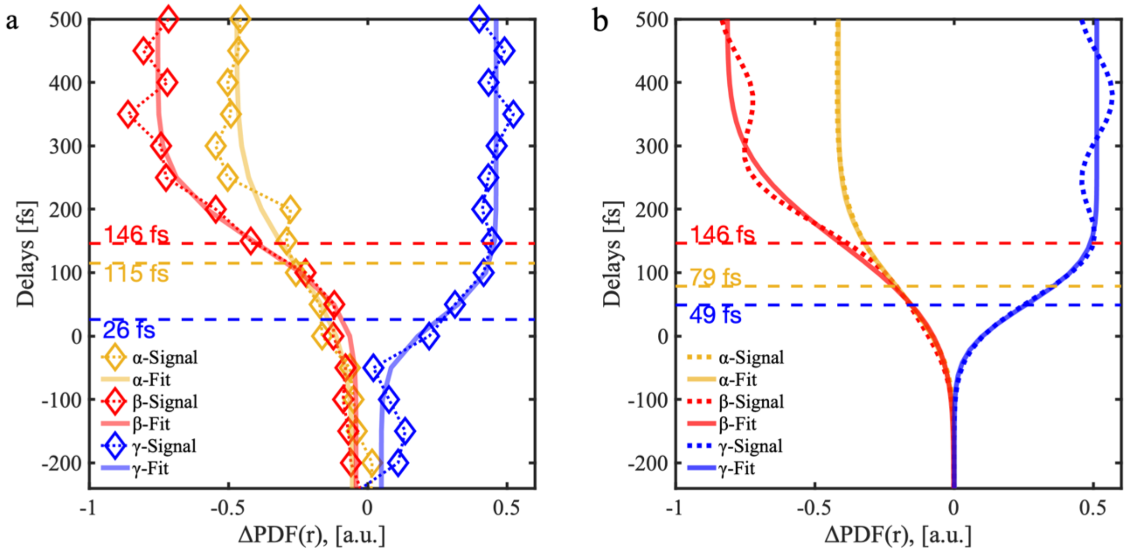

Supplementary Figure 8. Investigation of signal onset time at different pair distances. In this figure, we show the extracted signal onset time of α, β, and γ region for both the experiment (panel a) and simulation (panel b). We fit the signal with an error function at each region, respectively. The horizontal dashed lines in both panels indicate the onset times with respect to time zero. The corresponding onset time values are labeled.

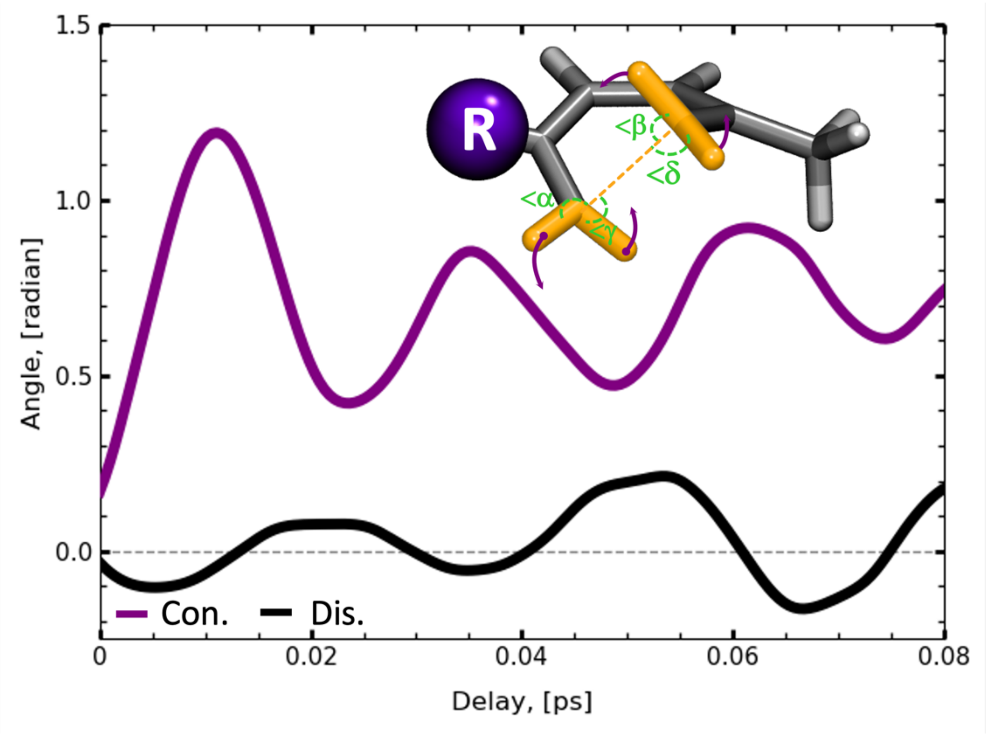

Supplementary Figure 9. The Averaged Conrotatory and Disrotatory Angles of αTP on S_1. The direct average for the conrotatory and disrotatory angles from all 60 ICs in the first 80fs of propagation on S_1. The conrotatory angle increased from around 0 to >1.0 radians while the disrotatory angle remained low. The conrotatory and disrotatory angles are defined as $<\alpha + <\delta - <\beta - <\gamma$ and $<\alpha + <\beta - <\delta - <\gamma$, respectively, and are shown in the inset. The purple sphere represents the isopropyl group without any specific orientation.

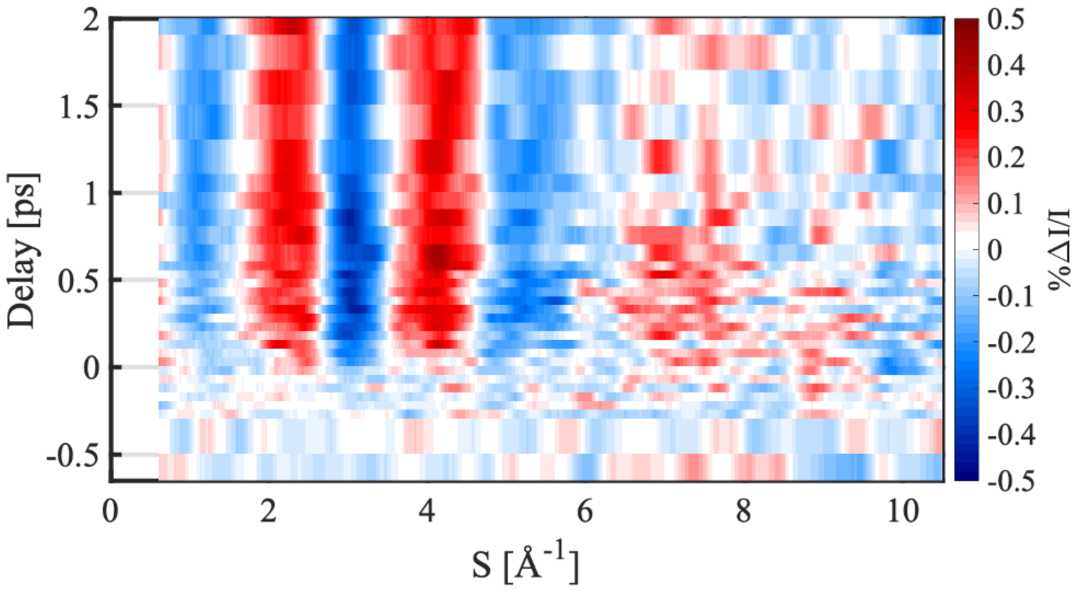

Supplementary Figure 10. Experimental time-dependent diffraction percentage difference signal. The false colormap intensities are shown as a percentage difference signal.

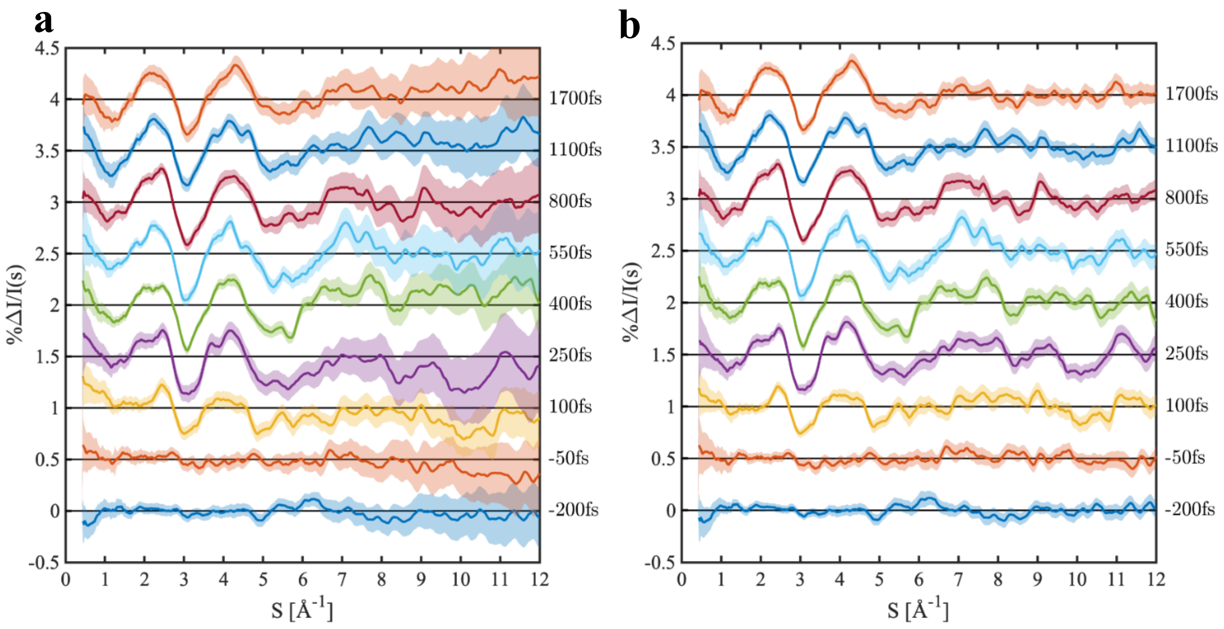

Supplementary Figure 11. Low-order polynomial background removal of the diffraction signal. Panel **a:** Diffraction percentage difference signal (%ΔI/I) at different delays plotted as colored lines. The corresponding shaded areas reflect the error bar (1 standard deviation). Panel **b:** Analogous plot to panel **a**, but after the low-order polynomial background removal process. The black lines indicate the baseline of each curve. The delay information is labeled on the right.

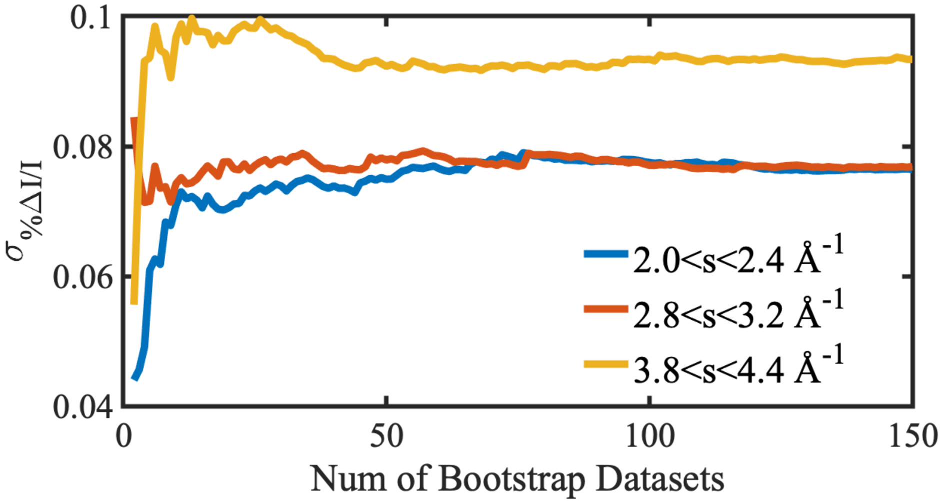

Supplementary Figure 12. Statistical uncertainty development of the bootstrapping analysis. The uncertainty as a function of the number of bootstrapping datasets used in the analysis. One standard deviation of the diffraction percentage difference signal at several different s regions are reflected. The uncertainty undergoes a saturation after 100 bootstrapping datasets.

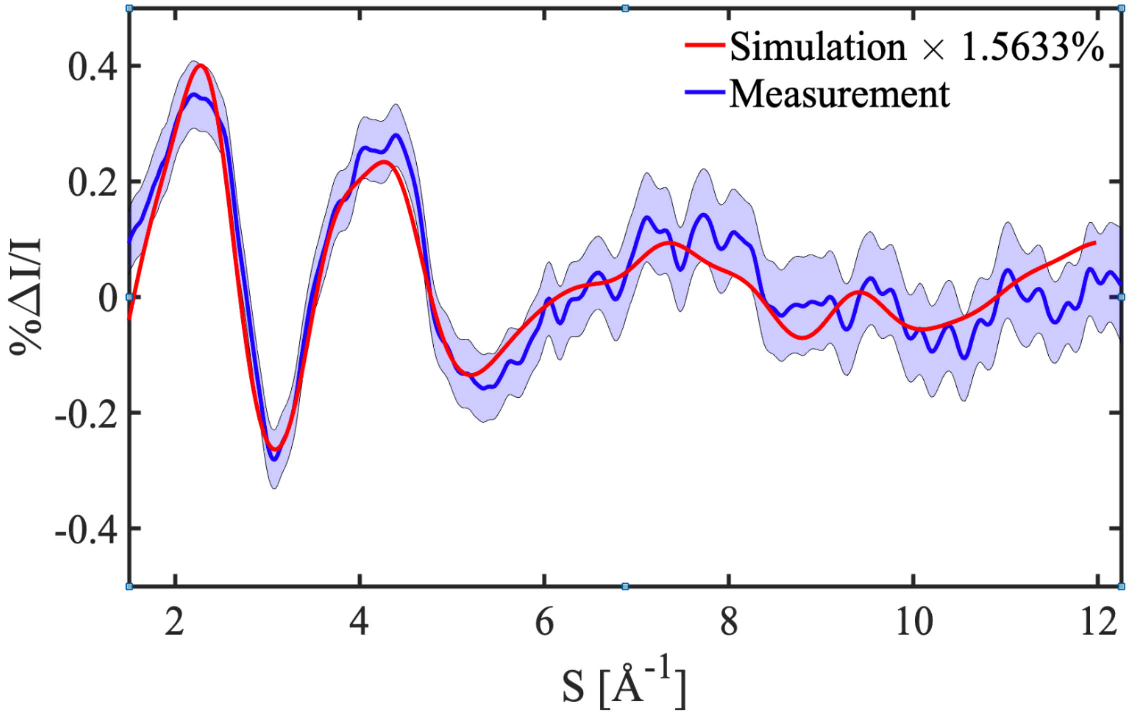

Supplementary Figure 13. Experimental and simulated percentage difference of the diffraction signal. In this figure, two curve plots show the experimental signal and simulated percentage difference signals at 1 ps delay. The simulation is scaled by a factor of 0.0156 to match the amplitude of the experiment. This suggests an excitation ratio of γ = 1.56%. The shaded blue areas represents the standard deviation of the experimental diffraction signal.

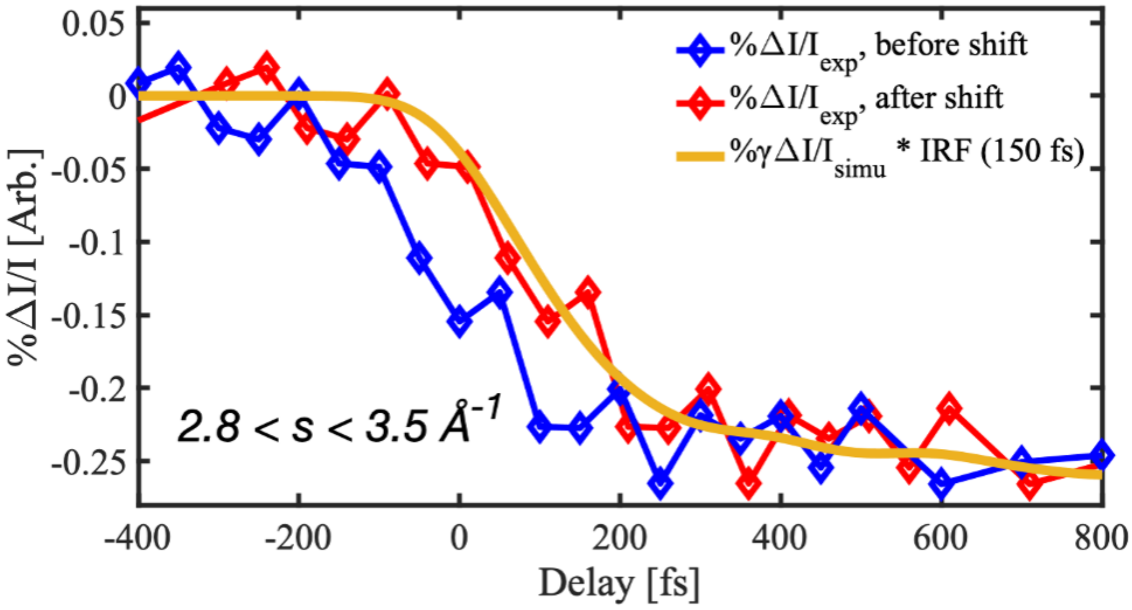

Supplementary Figure 14. Determination of the exact time-zero of the experiment. Both the simulation and experimental data are integrated between 2.8 and 3.5 $Å^{-1}$. The simulation is convolved with a Gaussian function of FWHM of 150 fs.

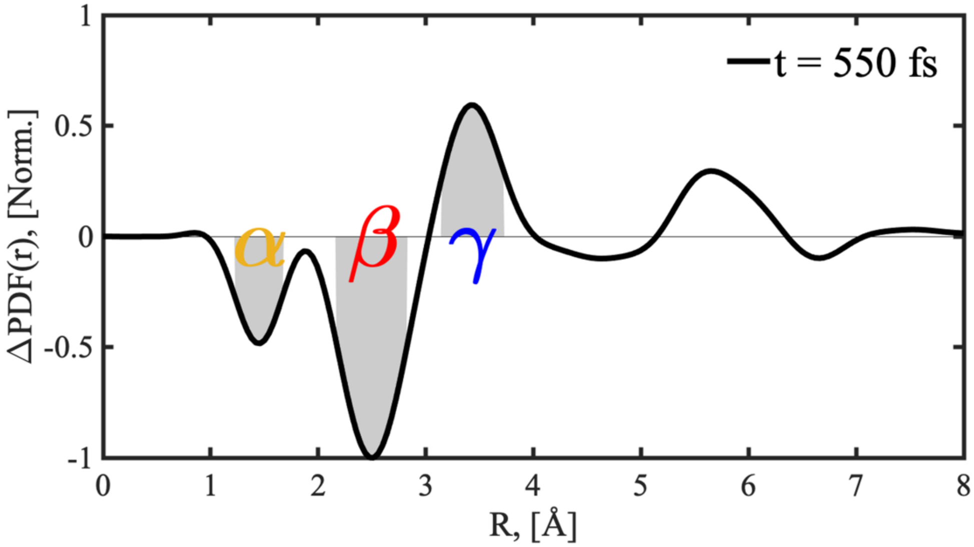

Supplementary Figure 15. Determination of the areas corresponding to different integration ranges of Fig. 3. The line in the figure shows the averaged ΔPDF(r) at t = 550 fs of all the three rotamers, whereas the gray plots indicated the areas' limits of interest corresponding to the carbon coordination spheres. The areas are chosen according to the FWHM of each peak or trough of the simulated ΔPDF(r).

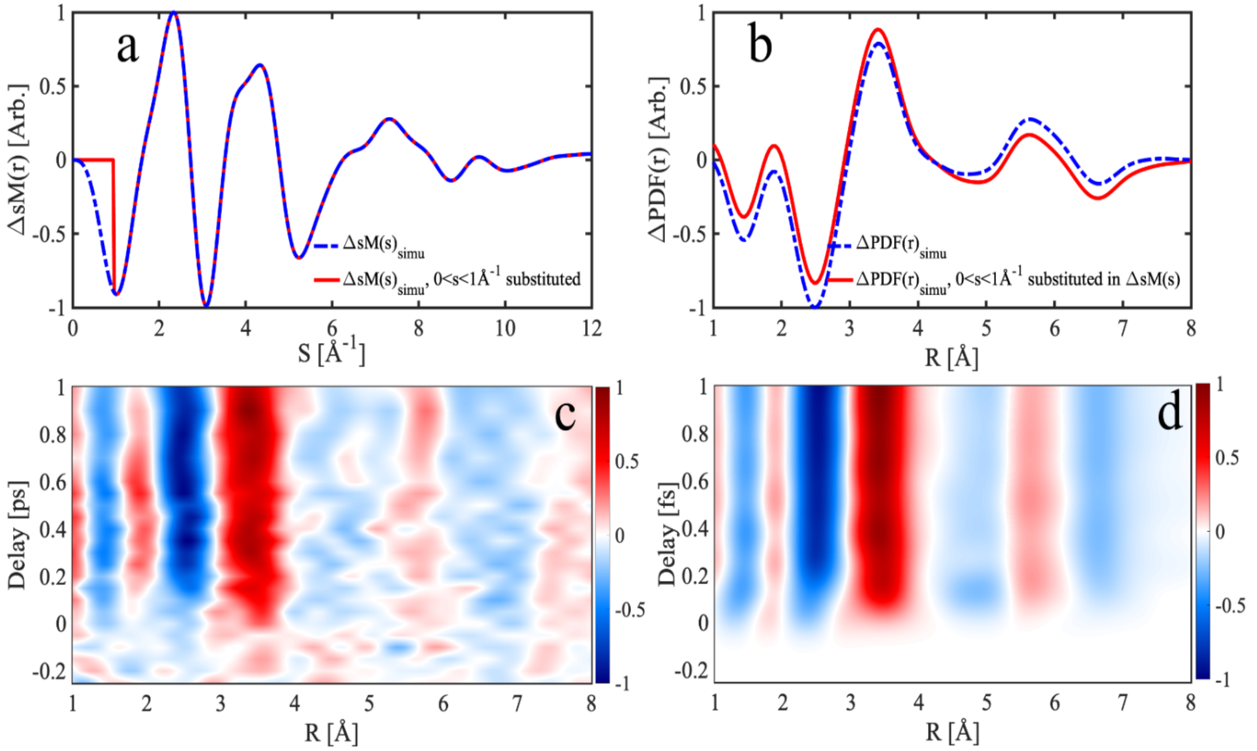

Supplementary Figure 16. Investigation of the low s signal in the calculation of the pair distribution function. In this figure, we show the influence of the low s signal in calculation of the pair distribution function. Panel **a** shows the simulated ΔsM(s) signal around 500 fs. The blue dashed curve reflects the simulation, whereas the red curve reflects the low s signal substituted by zeros. Panel **b** shows the ΔPDF(r) based on the signals in panel **a**. Panels **c** and **d** plot the time-dependent ΔPDF(r) of experiment and simulation with the low s signal replaced by zeros.

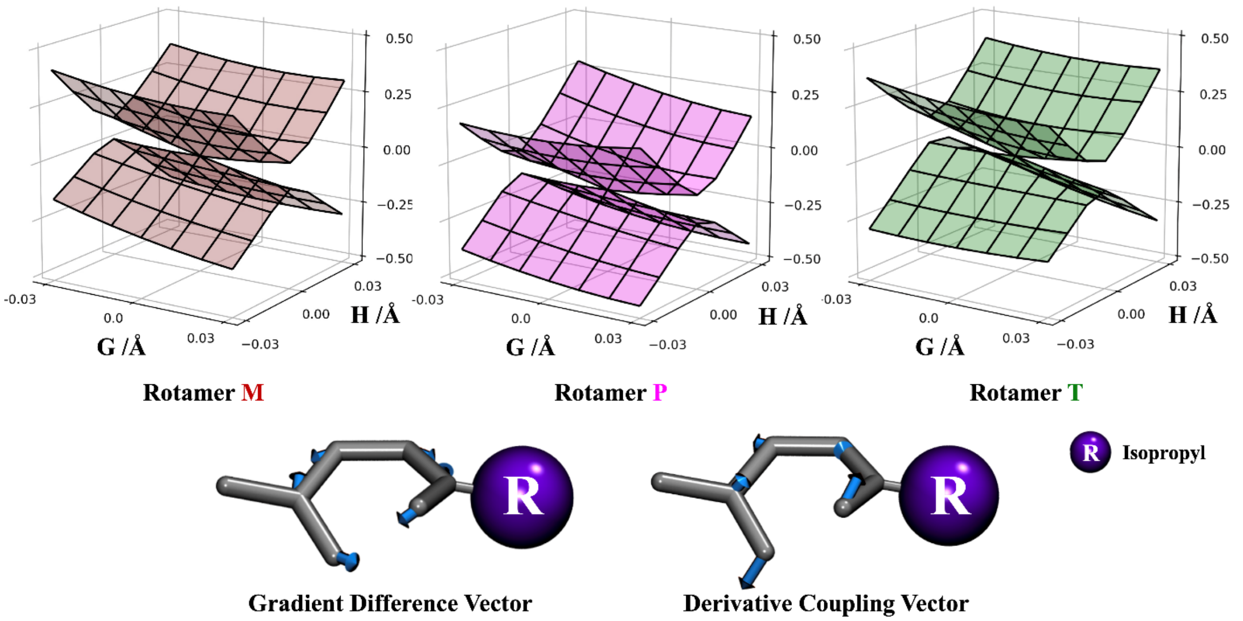

$$\mathbf{G} = \frac{1}{2}\frac{\partial}{\partial R}(E_I(R) - E_J(R))$$

$$\mathbf{H} = \left\langle \psi_I \middle| \frac{\partial}{\partial R} \middle| \psi_J \right\rangle$$

Supplementary Figure 17. The Branching Space at the Ring-Open S_0/S_1 MECI for the M, P, and T Rotamers. The branching space at the ring-opening S_0/S_1 MECI geometry for rotamers M (red), P (pink), T (green). The gradient-difference vector (left) and coupling vector (right) are shown on the ring-open MECI geometry with blue arrows. The purple sphere represents the isopropyl group without any specific orientation. All rotamers show a peak-like topography at the ring-open MECI point like CHD and αPH.

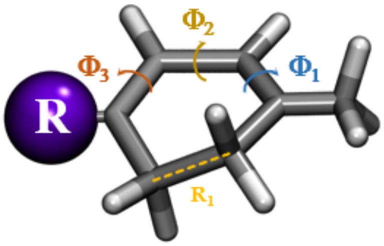

Conformer	R_1 (Å)	$\|\Phi_1\|$ (°)	$\|\Phi_2\|$ (°)	$\|\Phi_3\|$ (°)
αTP	≤ 1.8	≤ 80	≤ 80	≤ 80
cZc-IPMHT	> 1.8	≤ 80	≤ 80	≤ 80
cZt/tZc-IPMHT	> 1.8	≤ 80 or ≥ 100	≤ 80	≥ 100 or ≤ 80
tZt-IPMHT	> 1.8	≥ 100	≤ 80	≥ 100

Supplementary Figure 18. Classifying αTP and it IPMHT Isomers on S_0. Three angles and one distance are utilized to define the geometries of the isomers. In addition to αTP, three different ring-opening isomers are identified, cZc, cZt/tZc, and tZt-IPMHTs. As shown by the molecular cartoon in the top, three dihedral angles, Φ_1, Φ_2, Φ_3 across the CHD ring structure and distance R_1 between C_3-C_4. In the table, each row corresponds to the four classification criteria used to bin geometries from the ground state TBF trajectories.

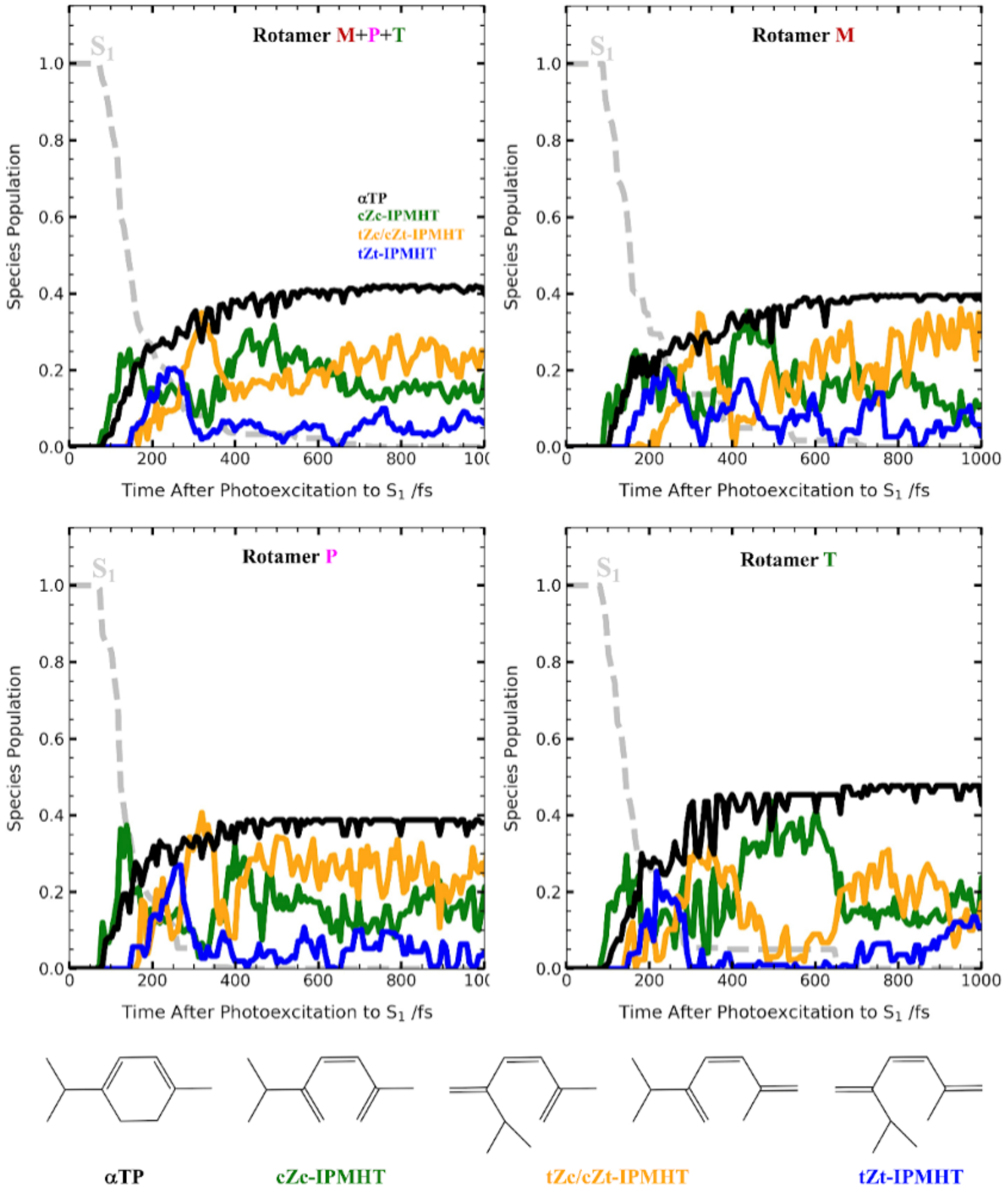

Supplementary Figure 19. Population of αTP and ring-opening product isomers on the S_0 after excitation. The percentage of the ground -state population binned into IPMHT isomers from optimized ground-state geometries via torsional angles Φ_1, Φ_2, Φ_3, and R_1 (see **Supplementary Figure 18**). Time zero corresponds to the spawn time of each TBF to S_0. Panels **a** to **d** show the relative population of the different product isomers as a function of time as well as the excited state population. Panel **a** reflect the population of all the three-ground state rotamers, whereas panels **b** to **d** showcase the populations from the individual ground state rotamers. The cartoon diagrams of the geometries are depicted in the bottom of

TABLES

Pair distance	α		β		γ	
	**Exp.**	**Simu.**	**Exp.**	**Simu.**	**Exp.**	**Simu.**
t_0	115 (23)	79 (0.2)	146 (11)	146 (1)	26 (15)	49 (1.3)
τ	252 (74)	213 (0.6)	186 (35)	257 (3.5)	48 (48)	130 (4.0)

Supplementary Table 1. Fitted parameters of the error function fit of the signal onset time and the width. As shown in Equ. 13, t_0 and τ are corresponding to the shift (signal onset time) and the width in the error function. The values in the parentheses indicate the uncertainty of the fits by taking 68% confidence. All the values are in units of femtoseconds. The fitted signal can be viewed in **Supplementary Figure 8** with the signal onset time labeled.

	S_0/S_1 CI (Open)			S_0/S1 CI (Closed)		
**Isomer**	Total	αTP	cZc-IPMHT	Total	αTP	cZc-IPMHT
Total	100 +/- 0	42 +/- 4	58 +/- 4	0	-	-
Rotamer M	100 +/- 0	40 +/- 8	60 +/- 8	0	-	-
Rotamer P	100 +/- 0	39 +/- 7	61 +/- 7	0	-	-
Rotamer T	100 +/- 0	48 +/- 7	52 +/- 7	0	-	-

Supplementary Table 2. Computational quantum yield for ground state rotamers through the Opening and closed CIs. Two types of CIs (Open and Closed) are identified. As shown in the table, all the transitions from the excited state to the ground state undergo the Ring-Opening CIs. The quantum yield for the αTP and product isomers are reflected with bootstrap uncertainties.

SUPPLEMENTARY REFERENCES

1. Ben-Nun, M. & Martínez, T. J. Direct Observation of Disrotatory Ring-Opening in Photoexcited Cyclobutene Using ab Initio Molecular Dynamics. *J. Am. Chem. Soc.* **122**, 6299–6300 (2000).
2. Yang, J. *et al.* Imaging CF3I conical intersection and photodissociation dynamics with ultrafast electron diffraction. *Science* **361**, 64–67 (2018).
3. Wolf, T. J. A. *et al.* The photochemical ring-opening of 1,3-cyclohexadiene imaged by ultrafast electron diffraction. *Nat. Chem.* **11**, 504–509 (2019).
4. Yang, J. *et al.* Simultaneous observation of nuclear and electronic dynamics by ultrafast electron diffraction. *Science* **368**, 885–889 (2020).
5. Champenois, E. G. *et al.* Conformer-specific photochemistry imaged in real space and time. *Science* **374**, 178–182 (2021).
6. Ihee, H., Goodson, B. M., Srinivasan, R., Lobastov, V. A. & Zewail, A. H. Ultrafast Electron Diffraction and Structural Dynamics: Transient Intermediates in the Elimination Reaction of $C_2F_4I_2$. *J. Phys. Chem. A* **106**, 4087–4103 (2002).
7. Feenstra, J. S., Park, S. T. & Zewail, A. H. Excited state molecular structures and reactions directly determined by ultrafast electron diffraction. *J. Chem. Phys.* **123**, 221104 (2005).
8. Park, S. T., Feenstra, J. S. & Zewail, A. H. Ultrafast electron diffraction: Excited state structures and chemistries of aromatic carbonyls. *J. Chem. Phys.* **124**, 174707 (2006).
9. Salvat, F., Jablonski, A. & Powell, C. J. ELSEPA—Dirac partial-wave calculation of elastic scattering of electrons and positrons by atoms, positive ions and molecules. *Comput. Phys. Commun.* **165**, 157–190 (2005).

10. Thompson, A. L., Punwong, C. & Martínez, T. J. Optimization of width parameters for quantum dynamics with frozen Gaussian basis sets. *Chem. Phys.* **370**, 70–77 (2010).

11. Makhov, D. V., Glover, W. J., Martinez, T. J. & Shalashilin, D. V. Ab initio multiple cloning algorithm for quantum nonadiabatic molecular dynamics. *J. Chem. Phys.* **141**, 054110 (2014).
