# Supplementary material for: Rehybridization dynamics into the pericyclic minimum of an electrocyclic reaction imaged in real-time
Source: Nat Commun. 2023 May 18;14:2795. doi: 10.1038/s41467-023-38513-6 (PMC10195774; doi:10.1038/s41467-023-38513-6)
Supplement: Supplementary file 3 — Description of Additional Supplementary Files [file 41467_2023_38513_MOESM3_ESM.pdf]

Description of Additional Supplementary Files

File Name: Supplementary Data 1
Description: Geometries, energies, and CI vectors of stationary points on the potential energy surface of α-terpinene

File Name: Supplementary Movie 1
Description: Animated version of Figure 5. The movie shows the full evolution two-dimensional projections of the simulated excited state wavepacket density over the first 200 fs after optical excitation. The top plot shows the projections (red contours) onto the (C_3-C_4) distance and the conrotatory deplanarization angle ψ from Fig. 4c. An Analogous projection onto the (C_3,C_{10}) distance and ψ are depicted in the bottom plot. For comparison, the α and γ areas of Fig. 3 are marked in yellow and blue. Additionally, the geometries at which population transfer to the ground state ultimately takes place are shown as grey circles with sizes proportional to the relative amount of transferred population. The minimum energy conical intersection geometry is marked as a green circle and the geometry of the pericyclic minimum as a red cross.
