# Supplementary figures and images for: Rehybridization dynamics into the pericyclic minimum of an electrocyclic reaction imaged in real-time
Source: Nat Commun. 2023 May 18;14:2795. doi: 10.1038/s41467-023-38513-6 (PMC10195774; doi:10.1038/s41467-023-38513-6)

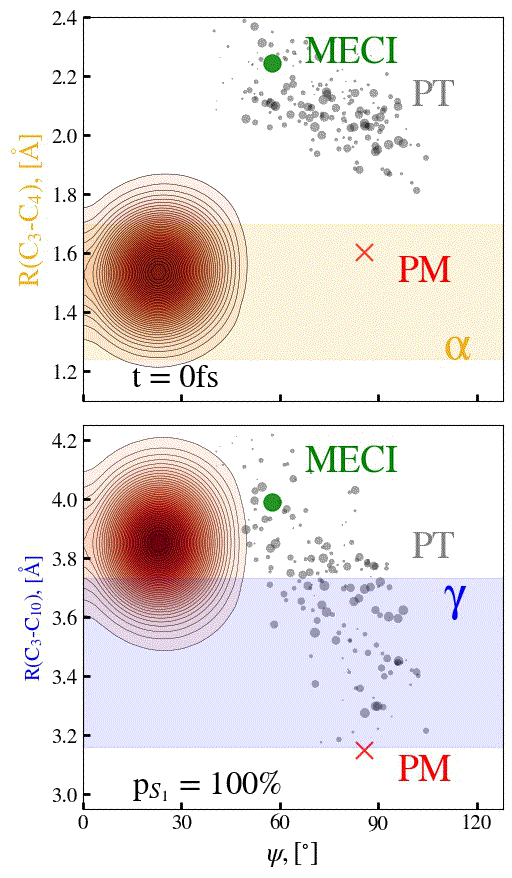

Supplement: Supplementary file 5 — Supplementary Movie 1 [file 41467_2023_38513_MOESM5_ESM.gif]
